# Coverage and Socio-Economic Inequalities in Breast Cancer Screening in Low- and Middle-Income Countries: Analysis of Demographic and Health Surveys Between 2010 and 2019

**DOI:** 10.1200/GO.22.65000

**Published:** 2022-05-05

**Authors:** Derrick Bary Abila, Grace Kangoma, Ruth Ketty Kisuza, Sulaiman Bugosera Wasukira, Provia Ainembabazi, Henry Wabinga

**Affiliations:** ^1^ College of Health Sciences, Makerere University, Kampala, Uganda; ^2^ School of Public Health, Makerere University, Kampala, Uganda; ^3^ School of Biomedical Sciences, Makerere Uiversity, Kampala, Uganda; ^4^ Infectious Disease Institute, Kampala, Uganda

## Abstract

The World Health Organisation (WHO) launched the Global Breast Cancer Initiative (GBCI) in 2020 intending to reduce global breast cancer mortality by 2.5% per year until 2040, thereby averting an estimated 2.5 million deaths. In this study, we aimed to determine the coverage and socio-economic inequalities in the screening for breast cancer over one decade before the establishment of the GBCI.

**METHODS:**

For each country, using STATA 16 software and sampling weights, we analyzed the datasets of Demographic and Health Surveys (DHS) that included questions on breast cancer screening and were conducted between 2010 and 2019 in low- and middle-income countries. We included women aged 15-49 years and considered screening using breast self-examination (BSE), clinical breast examination (CBE), and mammography. Absolute and relative inequalities were determined using the Slope Index of Inequality (SII) and Concentration Index (CIX) respectively.

**RESULTS:**

A total of 18 surveys from 13 countries were included in this study. Only six surveys from five countries measured the rates of screening by mammography which ranged from 5.58% to 12.96%. Considering screening using any method, the proportion that had ever screened for breast cancer ranged from 2.53% to 60.21%. Higher rates of screening were seen in upper-middle-income countries compared to low-income countries. For the CIX for screening using any method, the inequalities were pro-rich in all the countries except the Philippines where it was pro-poor with a CIX of –2.84 (*P* value .015). For the CIX and SII for screening using mammography, the inequalities were pro-rich in all the countries.

**CONCLUSION:**

There exist socio-economic disparities in the coverage of breast cancer using mammography, clinical breast examination, and breast self-exam. There is a need to address these disparities to achieve the targets of breast cancer control by the GBCI.

